# The relationship between student’s perceptions of their school environment and academic achievement

**DOI:** 10.3389/fpsyg.2022.959259

**Published:** 2023-02-01

**Authors:** Edward Edgerton, Jim McKechnie

**Affiliations:** Division of Psychology, School of Education and Social Sciences, University of the West of Scotland, Paisley, United Kingdom

**Keywords:** physical learning environment, academic achievement, subjective and objective data, attendance, socioeconomic status, gender, school behavior

## Abstract

Research within an educational context has demonstrated the importance of variables such as socioeconomic status, gender and school attendance as predictors of academic achievement, however research investigating the role of the physical learning environment on academic achievement is more limited and what research has been conducted often focuses on objective characteristics such as temperature, air quality and noise. In contrast this study measures students’ subjective perceptions of their physical school environment and explores how these perceptions along with socioeconomic status, gender and school attendance relate to academic achievement. In addition, we also examined a range of other important variables that could be potential mediating factors between environmental perceptions and academic achievement. The study was conducted with 441, S5 students in five secondary schools in Scotland. Students completed a questionnaire that measured their perceptions of their school environment, their behavior in school, and their learning goals. In addition, data on student academic achievement, attendance and socioeconomic status was provided by the Local Authority. Regression analysis indicates that students’ subjective perceptions of their physical school environment, along with attendance, socioeconomic status and gender are all significantly related to academic achievement. In addition, subsequent analysis indicates that the relationship between students’ subjective perceptions of their physical school environment and academic achievement is mediated by important “in-school behaviours,” namely engaging behavior and environmental difficulty. The implications of these findings are discussed in terms of the direct and indirect relationship between student perceptions of their school environment and their academic achievement.

## 1. Introduction

Within an educational perspective, research has highlighted a range of factors that can, to a greater or lesser extent, predict student academic achievement. These include student characteristics such as motivation (Pekrun et al., 2017) and self-efficacy (Zimmerman, 2000); school climate (Steinmayr et al., 2018); socioeconomic status (SES) (Liu et al., 2020); gender (Ghazvini and Khajehpour, 2011); parental education (Hotz and Pantano, 2015); and attendance (Gottfried, 2014). In addition, there is also a growing body of research suggesting that the physical learning environment of schools can also predict a range of educational outcomes including academic achievement (Barrett et al., 2015). This paper extends this research by examining how students’ subjective perceptions of their school environment relate to their academic achievement relative to other previously established variables such as SES, gender and attendance.

In comparison with issues such as pedagogical approaches, the role of the physical learning environment within teaching and learning has received less attention within educational research. However, from an environmental psychology perspective, research has demonstrated how physical characteristics of the learning environment are related to a range of student educational outcomes and experiences. These include: student attitudes such as the desire to go to school and a feeling of pride in their school (Rudd et al., 2008); problem behavior such as truancy and drug use (Kumar et al., 2008); students feelings of safety within schools (McEwen et al., 2007); and greater use of “positive” approaches to learning (Edgerton et al., 2011). However, given the focus of this paper, it is pertinent to review research on the impact of physical characteristics of the learning environment on academic achievement.

Relevant characteristics of the physical learning environment can include both psychosocial features and physical features. Research on psychosocial features have shown how the amount of space per child in a classroom (spatial density) can be as important as the number of children in a classroom (class size), and that less space per child negatively impacted on girls’ academic achievement and boys’ classroom behavior (Maxwell, 2003). Maxwell and Chmielewski (2008), conducted research on classroom personalization and found that attempts to increase the level of student classroom personalization by increasing the amount of student work on display and involving students in designing displays, was shown to enhance the self-esteem of first grade students in an elementary school (Maxwell and Chmielewski, 2008).

Research focusing on specific physical features have shown the importance of lighting within schools, with students under full-spectrum fluorescent lamps with ultraviolet supplements having better educational achievement than students under other artificial lighting conditions (Hathway, 1995). A meta-analysis conducted by Pilcher et al. (2002) concluded that temperature extremes negatively impact performance on a wide range of cognitive-related tasks, with temperatures above (32°C) or below (10°C) resulting in the greatest decrement in performance in comparison to neutral temperature conditions (14.88% decrement and 13.91% decrement, respectively). Research on ventilation levels within schools showed that low (poor) ventilation within classrooms significantly reduced students’ attention and negatively affected memory and concentration (Bakó-Biró et al., 2012). Acoustical aspects of learning environments have also been shown to impact on cognitive task performance with “classroom-babble” (noise by children alone) impairing performance on verbal tasks and “classroom babble plus environmental noise” impairing speed of processing tasks (Dockrell and Shield, 2006). A comprehensive, literature review of learning environments and student educational outcomes and experiences, highlighted the effect of physical characteristics of the learning environment, such as air quality and noise, on learning (Higgins et al., 2005). The researchers concluded that while there was clear evidence of a positive effect where the learning environment is brought up to a minimum standard (by improving these characteristics), there was less evidence to support “going beyond” this minimum standard. This view was echoed by Earthman (2004) who argued that while inadequate school buildings contribute to poor student performance, schools might not need to be any more than adequate. Interestingly, Higgins et al. (2005) go on to argue that improving school environments may have less to do with changing specific physical characteristics and more to do with how the change process is managed, in particular attempts to engage with, and involve users.

While this research suggests the importance of different environmental characteristics within learning environments, it also includes some limitations. Firstly, while the outcomes often include performance on important cognitive tasks and abilities such as speed of processing, concentration, and memory, there are relatively few studies that demonstrate how the physical learning environment impacts on student academic achievement. One noticeable exception to this, was a study conducted in primary schools in England by Barrett et al. (2015) which found that differences in levels of stimulation, individualization and naturalness within classrooms, explained 16% of the variance in student learning progress throughout a year. Findings such as these are rare and indicate that it is possible to quantify the impact of the learning environment on student performance and that this impact can be significant.

A second limitation with the previously cited research is the focus on “single-variable” studies. While these have improved our understanding of how specific aspects of a learning environment may impact on students, they do not recognize that the student experience is rarely determined by a single factor but is instead influenced by the combination of multiple factors. This has led to attempts to investigate learning environments that focus on the summative experience of all aspects of the physical learning environment whether this is at the classroom level (Barrett et al., 2015) or at the whole school level (Kumar et al., 2008). This approach reflects a fundamental characteristic of environmental psychology by recognizing that environment-behavior relationships should be viewed as holistic units.

A final limitation with much of the research cited above is the focus on the direct effects of the physical environment using objective measures such as CO2 levels (air quality) decibel levels (acoustics) and temperature levels (thermal comfort). While this approach supports an understanding of causal relationships between physical characteristics of the learning environment and student outcomes, it does not consider the potential indirect or symbolic effect of the physical environment (Weinstein and Woolfolk, 1981), and does not recognize the importance of individual differences and students’ subjective experience of their physical learning environment. In contrast, a number of researchers have argued for the need to assess students’ perceptions of their learning environments. Using an online survey, Zheng Yang et al. (2013) measured university students’ perceptions of their classroom attributes and found that these perceptions were highly dependent on spatial characteristics such as visibility, furniture and ambient characteristics such as air quality and temperature. In addition, these researchers found that non-classroom factors such as gender, added valuable contextual information for understanding student perceptions of the classrooms. For example, female students were generally more positive about temperature and visibility and less positive about acoustics than male students. Zheng Yang et al. (2013) go on to argue that attempts to improve learning environments must recognize the importance of students subjective perceptions.

The idea that the learning environment might function on a symbolic level is not new (Proshansky and Wolfe, 1974) and researchers such as Weinstein and Woolfolk (1981) have argued that the physical design of learning spaces can be seen as a source of information that influence student expectations and that research should explore this in more detail by looking at how students’ inferences about environmental characteristics relate to measures of student educational outcomes and experiences. For example, Sommer (1977) explains how the physical arrangement of classroom furniture combined with real and symbolic barriers, indicate to students the level of classroom interaction that is preferred.

In a similar vein, Maxwell (2007) demonstrated that learning spaces that support the development of competence in children are likely to enhance self-esteem, and that demonstrating a link between the learning environment and self-esteem may be an important step in explaining how the physical learning environment may relate to academic achievement. For example, self-esteem may impact on student motivation which in turn impacts on academic achievement (Maxwell, 2007). In this respect, student characteristics such as motivation may be seen as a mediator in the relationship between the physical learning environment and academic achievement. A study on new secondary school buildings in Scotland found that these new schools were associated with higher ratings of security from students (Edgerton et al., n.d.). Within the context of the Scottish education system, this finding is significant as the national curriculum in Scotland emphasizes the importance of schools promoting an atmosphere of safety and security for young people (The Scottish Government, 2008). With respect to the relationship between the learning environment and academic achievement, feelings of safety and security might be seen as a potential mediator variable, i.e., if students feel safe and secure in their school, they may be more likely to focus on learning activities associated with academic achievement.

A number of researchers have also recognized that students are able to “read” the physical environment of their school and how this relates to them, and that the physical learning can communicate the school or even society’s values to students (Maxwell, 2003; Fine et al., 2004). For example, if the learning environment communicates that children are a low priority, e.g., through poor maintenance or outdated facilities, the message is negative.

The preceding review suggests that the physical learning environment can be an important source of information for students and the way in which this is perceived can relate to characteristics such as student motivation and engagement which in turn may be related to academic achievement. From a theoretical perspective the bioecological model proposes that learning and psychological functioning are influenced by multiple, nested layers of the context of the individual (Bronfenbrenner and Morris, 2006) however, one neglected aspect of this model is the built environment (Evans, 2003). The relationship between the physical learning environment, psychological states (such as motivation), individual perceptions of the environment and academic achievement, has received less attention in the empirical literature and particularly within recent times.

In addition to understanding students’ subjective perceptions of their learning environment, research has also shown the importance of understanding students’ subjective perceptions of their own behavior within school. For example, in relation to educational outcomes, Edgerton et al. (2011) found that secondary school students with more positive perceptions of their school environment were more likely to perceive themselves as engaging more with school (e.g., volunteering within class) and having higher levels of self-esteem. Likewise, Midgely (2002) measured students’ perceptions of their motivational orientation through the development of the Patterns of Adaptive Learning Scales (PALS) that assessed students’ subjective perceptions of their personal achievement goals and their perceptions of the goal structure in the classroom. Research using this measure with students has shown how perceptions of learning goals may be associated with subjective perceptions of wellbeing and that this might be influenced by cultural factors (Tian et al., 2017).

This paper addresses the above limitations by using a subjective measure of students’ perceptions of their “whole” school environment and explored how this is related to academic achievement using national, standardized measures of academic achievement. In addition, we included measures of relevant educational outcomes and experiences, and secondary data on other variables that research has consistently shown to be important predictors of academic achievement (i.e., SES, attendance and gender).

The primary aim of this study was to understand if students’ subjective perceptions of their school environment were related to academic achievement alongside previously identified variables. The secondary aim was to investigate whether any relationship between students’ perceptions of their school environment and academic achievement is mediated by other potential factors. The hypotheses for the study are therefore:

*H1*: Students’ subjective perceptions of their physical school environment will be significantly related to academic achievement along with SES, attendance and gender.

*H2*: The relationship between students’ subjective perceptions of their physical school environment and academic achievement will be mediated by student perceptions of their engaging behavior, environmental difficulty, security, and motivation.

## 2. Methodology

### 2.1. Background

The data for this study was collected as part of larger-scale study evaluating a secondary school building program with a Local Authority in Central Scotland. In Scotland such schools accommodate students between 12 and 17 years of age. While the larger scale study collected data at different points in the construction process, for the purposes of this paper, we will only look at the data that was collected 46 months after the new schools had opened (May 2013). By adopting this approach, we focus only on those students that have had the most time in the new school environments without any of the inconvenience of the construction process.

### 2.2. Design

This study employed a correlational design using data that was collected using a specifically developed questionnaire to measure students’ perceptions of their school environment along with important education-related variables and their “in-school behaviour.” For the purposes of this study, the following data from the questionnaire was used.

### 2.3. Measures

#### 2.3.1. The physical school environment

Firstly, a measure of students’ perceptions of their physical school environment was created based on information obtained from a series of focus groups conducted with students in two of the schools involved in the school re-building program (for further details see McEwen et al., 2011). This section consisted of 60 items that covered different areas of the school, such as classrooms, social spaces, circulation spaces and toilet facilities, and were answered on a five-point Likert scale from “very poor” through to “very good.” The items showed good internal reliability (Cronbach’s alpha = 0.971) For the purpose of this study, we produced a student “global environmental perception” score (GEP) by summing the average score across these items. A copy of the 60 items can be found in Appendix 1.

#### 2.3.2. “In-school behaviours”

Secondly, a measure of important “in-school behaviours” was created and again this was based on information obtained from the series of focus groups. Three categories of behavior were identified, and students were asked to rate how often they performed a range of behaviors on a four point scale ranging from “never” through to “always.” The three categories of “in-school behaviour” were: Engaging Behavior (9 items), Environmental Difficulty (7 items), and Security (5 items). All of these sub-scales had acceptable levels of reliability as indicated by the following Cronbach’s alpha coefficients: Engaging Behavior (0.712), Environmental Difficulty (0.652), and Security (0.653). A copy of the “in-school behaviour” items can be found in Appendix 2.

#### 2.3.3. Achievement goal orientations

Thirdly, student motivation was measured using Midgely et al. (2000), Patterns of Adaptive Learning Scale (PALS). This measures the achievement goal orientations of students in relation to school achievement, i.e., how they approach, engage and evaluate their learning within an achievement context. PALS assesses three different learning goals: (i) Mastery Approach—this is considered a beneficial approach to learning where students desire to master the task at hand, (ii) Performance Approach—although there is some debate about whether this is a positive or negative approach to learning, it is generally considered to be a beneficial approach where students attempt to show that they can perform as well as or better than their peers, and (iii) Performance Avoidance—this is considered a maladaptive approach to learning where students avoid participating in class to avoid failing. As expected, there was good reliability for this standardized instrument, with the following Cronbach’s alphas: Mastery Approach (0.854), Performance Approach (0.846), and Performance Avoidance (0.696).

#### 2.3.4. Secondary data (gender, SES, and academic achievement)

Finally, in addition to the questionnaire data, the Local Authority provided the research team with secondary data for each student for the following variables: Gender; SES (based on postcode address—in Scotland these are ranked in SIMD deciles where 1 = highest level of deprivation and 10 = lowest level of deprivation); and Attendance level (ranging from 0 to 100%). Academic achievement was calculated based on validated data by the Scottish Qualifications Authority (SQA); this is the executive, non-departmental public body of the Scottish Government responsible for accrediting national educational awards. At S5 level, all courses have externally marked exams and students are awarded a band between 1 and 7 for each subject (with 1 being the highest band). To calculate a global score for students’ academic performance, the bands achieved for each course were multiplied by the appropriate value to reflect the level of difficulty as indicated by the SQA Credit Qualification Framework.

### 2.4. Participants

The study collected data from S5 students (approximately 16 years of age) in five schools. Once permission for the study was obtained from the Local Authority and ethical approval was granted by the University ethics committee, the questionnaires were administered in each school in either the assembly hall with all students from the year group present or in individual registration classes. In both cases, researchers were present during the data collection to distribute and collect the questionnaires and answer any questions that students might have.

If participants had missing data on any of the variables, they were excluded from the relevant analyses (in total, 18 participants eliminated, mostly due to lack of SIMD data). In total, across all schools, 441 students completed the questionnaire; this constitutes a response rate of 63.3%. Of these, 229 were male (51.9%) and 212 were female (49.1%).

### 2.5. Data analysis

Pearson correlational analyses were conducted to assess the relationship between the study variables. Multiple regression analysis was conducted to test the first hypothesis that GEP will be significantly related to academic achievement along with SES, attendance and gender. A hierarchical multiple regression analysis was then conducted to test the second hypothesis that the relationship between GEP and academic achievement will be mediated by student perceptions of their engaging behavior, environmental difficulty, security, and motivation.

## 3. Results

Table 1 displays the descriptive statistics for the main variables (with the exception of gender, which was explained in the methodology). GEP was normally distributed and higher scores indicate more positive perceptions of the physical school environment. Academic achievement and SES were slightly negatively skewed and since higher levels of SES indicate less deprivation, this group of students as a whole were from relatively affluent backgrounds. Attendance ranged from 1 to 100% and was heavily negatively skewed so a logit transformation was computed for subsequent analyses.

**Table 1:** Descriptive statistics for hypothesis one, study variables.

	Range	Mean ± Sd	Skewness ± SE
Global Environmental Perception (GEP)	123–278	204.34 ± 24.83	0.01 ± 0.12
Academic achievement	0–420	230.29 ± 96.51	−0.30 ± 0.12
SES	1–10	7.25 ± 2.91	−0.71 ± 0.12
Attendance	0.01–1	0.95 ± 0.07	−7.54 ± 0.12

Academic achievement scores were related to GEP, attendance, SES and gender (Table 2). There were moderate positive correlations between academic achievement and both attendance and SES and smaller positive correlations with GEP and gender (females scoring more highly than males). SES and attendance were also positively correlated but there were no other significant correlations. In particular GEP was not related to attendance, gender or SES.

**Table 2:** Correlations between study variables.

	Academic achievement	GEP	Attendance	SES
GEP	0.16^*^			
Attendance	0.29^*^	0.01		
SES	0.31^*^	0.03	0.14^*^	
Gender^†^	0.12^**^	0.04	−0.08	0.04

* *p* < 0.01,

** *p* < 0.05.

† Point biserial correlation.

A multiple regression was run to predict academic achievement from GEP, attendance, SES and gender. The multiple regression model significantly predicted academic achievement, *F*(4, 436) = 27.0, *p* < 0.001, adj. *R^2^* = 0.21. All four variables added statistically significantly to the prediction, *p* < 0.01. Regression coefficients and standard errors can be found in Table 3.

**Table 3:** Regression statistics for model with four predictor variables and academic achievement as outcome variable.

Academic achievement	*B*	95% CI for *B*	β	ΔR^2^	
Model					0.21^*^
Constant	−30.80				
Attendance	20.68^*^	13.89	27.51	0.26^*^	
SES	9.17^*^	6.35	11.99	0.28^*^	
Gender	28.00^*^	11.67	44.39	0.15^*^	
GEP	0.55^*^	0.22	0.88	0.14^*^	

B, unstandardized regression coefficient; CI, confidence interval; β, standardized coefficient. ΔR^2^, adjusted R^2^.

* *p* < 0.01.

Standardized beta values showed that attendance and SES were of similar importance with gender and GEP less important. However, it does indicate the GEP is a significant predictor of academic achievement and this supports the first hypothesis.

To test the second hypothesis, we conducted a further regression analysis that included potential mediating factors between GEP and academic achievement. These were the three categories of “in-school behaviours”: engaging behavior, environmental difficulty and security, and three learning goals (mastery approach, performance approach, and performance avoidance).

Table 4 gives the descriptive statistics for these variables. Engaging Behavior, Environmental Difficulty, Performance Approach, and Performance Avoidance were all normally distributed. Security was positively skewed and a transformation using the natural log was computed for subsequent analyses. Mastery approach was negatively skewed and so a transformation was computed for subsequent analyses.

**Table 4:** Descriptive statistics for hypothesis two, study variables.

	Range	Mean ± Sd	Skewness ± SE
Engaging behavior	12–35	24.00 ± 3.86	0.01 ± 0.12
Environmental difficulty	7–23	13.62 ± 2.79	0.54 ± 0.12
Security	5–17	7.75 ± 2.02	1.00 ± 0.12
Mastery approach	10–25	21.37 ± 3.16	−0.94 ± 0.17
Performance approach	5–25	13.00 ± 4.62	0.31 ± 0.17
Performance avoidance	4–20	11.46 ± 3.76	0.03 ± 0.17

Table 5 shows the partial correlations between GEP, “in-school behaviours” and learning goals controlling for attendance, SES and gender. GEP was positively correlated with engaging behaviors and mastery approach but was negatively related to environmental difficulty and security. Engaging behavior was strongly related to mastery approach and environmental difficulty was strongly related to security.

**Table 5:** Partial correlations between study variables.

	GEP	Engaging behavior	Environmental difficulty	Security	Mastery approach	Performance approach
Engaging behavior	0.305^*^					
Environmental difficulty	−0.403^*^	−0.039				
Security	−0.337^*^	−0.138^*^	0.302^*^			
Mastery approach	0.174^*^	0.349^*^	0.090	−0.001		
Performance approach	−0.052	0.104^**^	0.094	0.044	0.189^*^	
Performance avoidance	−0.085	0.067	0.099^**^	0.062	0.200^*^	0.738^*^

^*^p < 0.01; ^**^p < 0.05 attendance, SES and gender controlled.

A hierarchical regression analysis on academic achievement with attendance, SES and gender entered as predictors in the first block and GEP, engaging behavior, environmental difficulty, security, mastery approach, performance approach and performance avoidance entered stepwise in the second block was significant [*F*(5, 434) = 30.0, *p* < 0.01, adj. *R^2^* = 0.25]. The adjusted *R^2^* was 0.17 for the first block and 0.25 after the second block; the change of 0.08 was significant (*p* < 0.01).

There were five significant predictors, attendance, SES, gender, engaging behavior and environmental difficulty (Table 6, *p* < 0.01). Standardized beta values showed that attendance, SES and engaging behavior were of similar importance with gender and environmental difficulty less important. The model as a whole was a better fit than the earlier model with an adjusted *R^2^* of 0.25.

**Table 6:** Regression statistics for model with five predictor variables and academic achievement as outcome variable.

Academic achievement	*B*	95% CI for *B*		β	ΔR^2^
Model					0.25^*^
Constant	−7.55				
Attendance	16.82^*^	10.12	23.51	0.21^*^	
SES	8.35^*^	5.61	11.09	0.25^*^	
Gender	22.16^*^	6.02	38.30	0.12^*^	
Engaging behavior	6.37^*^	4.25	8.48	0.25^*^	
Environmental Difficulty	−4.21^*^	−7.05	−1.37	−0.12^*^	

B, unstandardized regression coefficient; CI, confidence interval; β, standardized coefficient. ^*^p < 0.01; ΔR^2^, adjusted R^2^.

**Figure 1:**
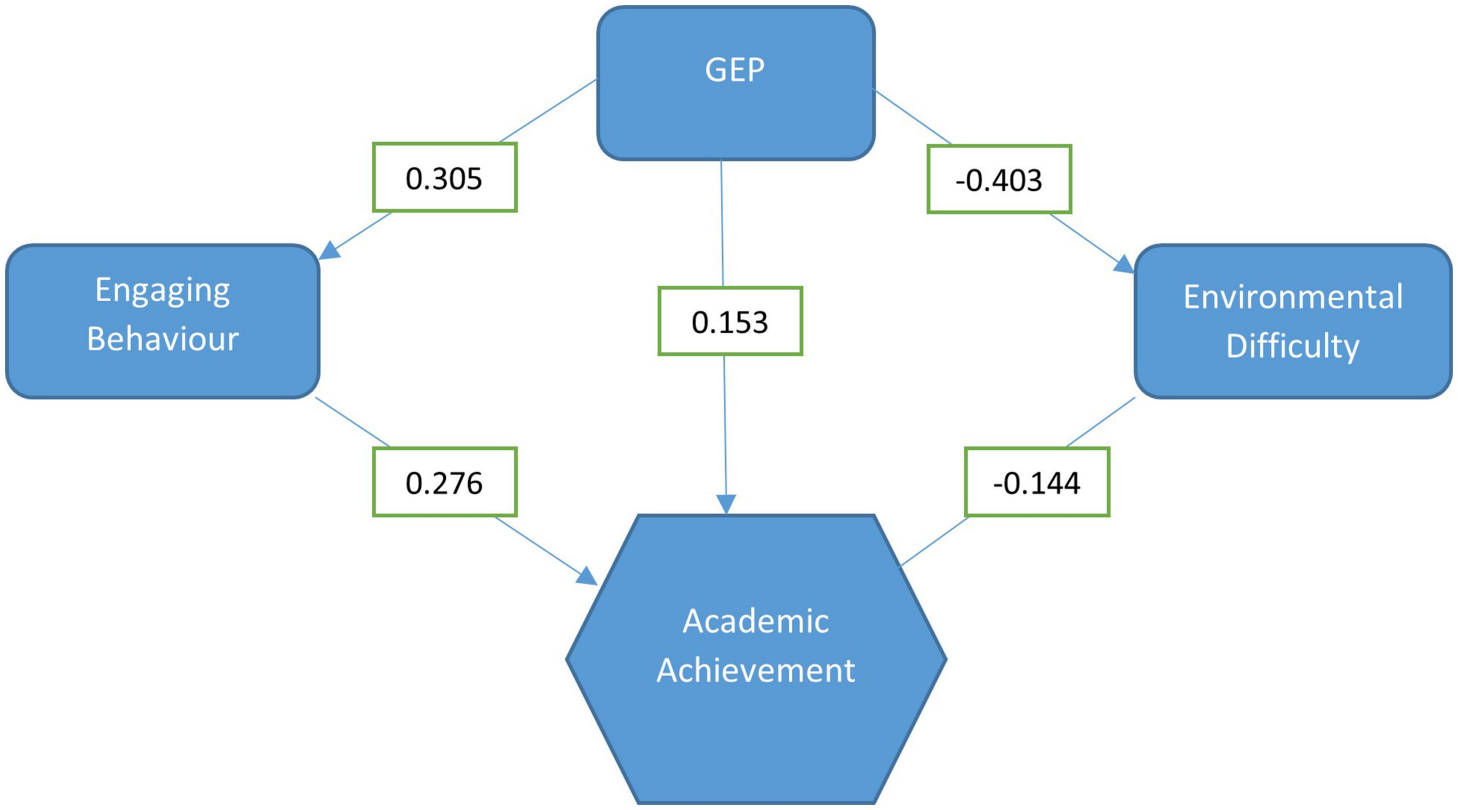
Relationship between global environmental perceptions and academic achievement.

Figure 1 shows the partial correlations between academic achievement, GEP, engaging behavior and environmental difficulty once attendance, SES and gender are controlled for. This indicates that while the direct correlation between academic achievement and GEP is modest, GEP is strongly related to both engaging behavior (positively) and environmental difficulty (negatively) which are both in turn related to academic achievement.

These results partially support Hypothesis 2 and indicate that the relationship between GEP and academic achievement is mediated by engaging behavior and environmental difficulty.

## 4. Discussion

The results support previous research that has highlighted that gender, SES, and attendance are significantly related with academic achievement. However, the results also make an important additional contribution by indicating that students’ perceptions of their physical school environment may also be important in explaining the variance in academic achievement. This finding suggests that the importance of the physical environment may depend on students’ perceptions of that environment (Vosko, 1984) and as such, should be treated as important factors to consider in attempts to improve learning environments (Siegel, 2003).

While the resulting model only explained 21% of the variance in academic achievement, it is worth noting that the model did not include a measure of IQ or general intelligence. Previous research has shown that measures of general intelligence are good predictors of academic achievement, with correlations between 0.5 and 0.8 (Deary et al., 2007; Rohde and Thompson, 2007) and psychometric measures of intelligence have been shown to account for about 30% of the variance of academic achievement, depending on level of education (Gustafsson and Balke, 1993; Roth et al., 2015). At the same time, these studies also highlight that a large part of the variance in academic achievement is not accounted for by measures of general intelligence and that for some students, there is a discrepancy between their actual and expected level of academic performance, given IQ is known; this is known as the “IQ-achievement gap” (Flynn, 1991). While this study measures academic achievement rather than general intelligence, it suggests that in addition to school attendance, SES and gender, students’ perceptions of their physical school environment may explain some of the discrepancy between actual and expected level of academic achievement.

In relation to the second aim of the study, the results offer partial support for the hypothesis as they show that the relationship between students’ perceptions of their physical learning environment and academic achievement may be mediated by certain “in-school behaviours” but not by learning goals. This suggests an indirect relationship between students’ perceptions of their physical learning environment and academic achievement with these perceptions being mediated *via* engaging behavior and environmental difficulty, i.e., more positive perceptions of the physical school environment are associated with more engaging behavior and less environmental difficulty, which in turn is associated with greater academic achievement. To better understand these results, it is useful to look in more detail at the concepts of engaging behavior and environmental difficulty as measured in this study.

Engaging behavior was assessed by asking students to indicate how often they performed behaviors such as using the library outside of class times, attending after school clubs or sports activities, volunteering for things when asked, helping other students in class with their work, and answering teachers’ questions in the classroom. We would argue that if students have more positive perceptions of their physical school environment then this symbolically indicates to students that their education is valued by important stakeholders in the education system in terms of investment, maintenance, etc. This is supported by the work of Weinstein and Woolfolk (1981) that highlights the potential symbolic effects of the physical environment. In addition, it is also likely that where students have more positive perceptions of spaces in their school environment such as classrooms and library and sports facilities, they are much more likely to perform “engaging behaviour” that relate to these spaces. This view could be interpreted using “person-environment fit theory” that has been developed in workplace studies; here “fit” is defined in terms of the comparison of person and environment characteristics to determine whether or not there is a match (Kristof-Brown and Billsberry, 2013). An important concept within this theory is that the fit between the person and the environment can be objective or subjective and subjective fit is based on the perception of the individual (Van Vianen, 2018). It is highly likely that greater levels of engaging behavior such as answering teachers’ questions in class, helping other students with their work and more use of library facilities could have a positive, direct impact on academic achievement as these are activities that support learning. However, it is also possible that greater levels of engaging behavior are associated with greater enjoyment or liking of school which in turn has been shown to relate to academic achievement (Ladd et al., 2000; Riglin et al., 2013) and a more positive school climate which is positively related to prerequisites for learning such as student engagement and attitudes toward school (Wang and Holcombe, 2010; Van Ryzin, 2011).

Environmental Difficulty was assessed by asking students to indicate the frequency with which they found it difficult to move around between classes because of the layout of the school, got confused with the layout of the school, and found different areas of the school too busy (corridors, toilets, social spaces, etc.). Here again, we can use “person-environment fit theory” to explain this finding as more positive perceptions of the physical school environment are likely to be associated with less subjective perceptions of environmental difficulty, i.e., students abilities and needs (person) have a better fit with physical characteristics of the school such as amount of space and legibility (environment). Using person-environment fit theory, we would argue that where students experience less difficulty interacting with their school environment, this facilitates activities that have positive consequences, e.g., being able to get to class on time without stress or difficulty puts the student in a frame of mind conducive to learning. Similarly, being able to find spaces that support peer interaction facilitates “positive” outcomes associated with informal learning and the importance of informal learning spaces has been demonstrated in other learning contexts (Acker and Miller, 2005).

Although the findings are promising, our study also has several limitations. First, the study was limited to one year group of students and we should be cautious in accepting the generalizability of our findings to other year groups of students. As Maxwell (2000) highlights, between group differences are important factors to consider when investigating users perceptions of their physical environment. A second limitation relates to our interpretation of causal relationships between GEP and academic achievement, i.e., it is possible that students who are doing well may perceive their school more positively and therefore, higher levels of academic achievement may lead to more positive environmental perceptions. A final limitation is that we used a global environmental perception measure that was based on a summative total of different areas of the school environment. However, it may be that different areas and spaces within the school environment might vary in importance to students and as such future, research might consider disaggregating the whole school environment into smaller units such as classrooms, circulation spaces and social spaces to examine the relative contribution of different areas of the school environment to students’ academic achievement. Adopting this approach might provide practical information of interest to stakeholders such as estate managers, design professionals and educational practitioners.

To summarize therefore, this study has demonstrated that students’ subjective perceptions of their physical school environment are related to academic achievement. In addition, this relationship is likely to be indirect with students’ subjective perceptions of their physical school environment being strongly associated with greater engaging behavior and less environmental difficulty which in turn is related to academic achievement.

These findings may have important implications for a range of stakeholders involved with school estates. Attempts to improve learning environments should recognize the value of understanding students’ subjective perceptions of their physical learning environments in addition to objective measures of important environmental characteristics; this includes symbolic aspects such as the message that the learning environment may be communicating to students. In addition, it is important to understand what aspects of their learning environment students perceive as being more important to them and how this might be influenced by individual differences between different groups of students such as age and gender.

## Data availability statement

The raw data supporting the conclusions of this article will be made available by the authors, without undue reservation.

## Ethics statement

The studies involving human participants were reviewed and approved by University of the West of Scotland, School of Social Sciences, Ethics Committee. Written informed consent to participate in this study was provided by the participants’ legal guardian/next of kin.

## Author contributions

EE and JM were responsible for the design of the study, the implementation of the study along with data analyses and dissemination. All authors contributed to the article and approved the submitted version.

## Funding

This research was partly funded by the East Dunbartonshire Council.

## Conflict of interest

The authors declare that the research was conducted in the absence of any commercial or financial relationships that could be construed as a potential conflict of interest.

## Publisher’s note

All claims expressed in this article are solely those of the authors and do not necessarily represent those of their affiliated organizations, or those of the publisher, the editors and the reviewers. Any product that may be evaluated in this article, or claim that may be made by its manufacturer, is not guaranteed or endorsed by the publisher.
