# Supplementary material for: The relationship between student’s perceptions of their school environment and academic achievement
Source: Front Psychol. 2023 Feb 1;13:959259. doi: 10.3389/fpsyg.2022.959259 (PMC9929545; doi:10.3389/fpsyg.2022.959259)
Supplement: Supplementary file 1 [file Data_Sheet_1.docx]

**Appendix 1**

List of items that formed the Global Environmental Perception (GEP) score

| **The outside of the school and the school grounds/playground** |
| --- |
| Attractiveness of the school building |
| Attractiveness of the school grounds/ playground |
| Amount of space |
| Places to sit |
| Comfortable places to sit |
| Choice of places to meet friends |
| Places to relax |
| **Places to meet with friends inside the school** |
| Attractiveness of meeting places |
| Amount of space |
| Places to sit |
| Comfortable places to sit |
| Choices of places to meet friends |
| Places to relax |
| **Corridors and Stairs** |
| Attractiveness |
| Amount of space |
| **Standard Classrooms** |
| Attractiveness |
| Ability to see the board |
| Ability to see the teacher |
| Ability to hear the teacher |
| Noise from outside *(N.B. very poor = disruption to the class)* |
| Temperature |
| Comfortable furniture |
| Ability to move around the class |
| Amount of space |
| Layout/arrangement of classroom |
| **Non-standard/ Practical Classrooms** |
| Attractiveness |
| Ability to see the board |
| Ability to see the teacher |
| Ability to hear the teacher |
| Noise from outside *(N.B. very poor = disruption to the class)* |
| Temperature |
| Comfortable furniture |
| Ability to move around the class |
| Amount of space |
| Layout/arrangement of classroom |
| **Toilet Facilities** |
| Attractiveness |
| Amount of space |
| Location of toilets |
| Number of toilets |
| **Indoor Sports Facilities** |
| Attractiveness of the changing rooms |
| Attractiveness of the Gym/Sports Hall |
| Temperature of Gym/Sports Hall |
| Ability to see the teacher |
| Ability to hear the teacher |
| Amount of space |
| Ability to move around |
| Suitability of Gym/Sports Hall for different sports |
| **Outdoor Sports Facilities** |
| Attractiveness |
| Number of outdoor pitches |
| Condition of outdoor pitches |
| Location of outdoor pitches |

**Appendix 2**

List of items that formed the ‘in-school behaviours’

| **Engaging Behaviour** |
| --- |
| I use the library outside of class times |
| I attend after school clubs or sports activities |
| I volunteer for things when asked |
| I try to work hard in my classes |
| I make new friends at school |
| I help others students in my class with their work |
| I get my homework in on time |
| I try to work hard in my classes |
| I answer the teachers' questions in the classroom |
| **Environmental Difficulty** |
| I find it difficult to move around between classes because of the layout of the school |
| I have to queue for the toilet |
| I am late for classes because the corridors are too busy |
| I think the school grounds are too busy |
| I get confused with the layout of the school |
| I think the places I meet with my friends inside the school are too busy |
| I think the dining hall/ canteen/ cafe is too busy. |
| **Security** |
| I see strangers inside the school building |
| I think it is easy for someone to steal my personal belongings |
| The school is vandalised |
| I avoid some areas of the school because I don't feel safe |
| I see strangers outside in the school grounds |
